# Prenatal diagnosis and pregnancy outcomes of 1492 fetuses with congenital heart disease: role of multidisciplinary-joint consultation in prenatal diagnosis

**DOI:** 10.1038/s41598-020-64591-3

**Published:** 2020-05-05

**Authors:** Xiuqing Qiu, Zongjie Weng, Min Liu, Xiujuan Chen, Qiumei Wu, Wen Ling, Hong Ma, Hailong Huang, Yuan Lin

**Affiliations:** 1 Department of obstetrics and gynecology, Fujian Maternity and Child Health Hospital, Affiliated Hospital of Fujian Medical University, 18 Daoshan Road, Gulou District, Fuzhou, Fujian, 350001 People’s Republic of China; 2 Department of Medical Ultrasonics, Fujian Maternity and Child Health Hospital, Affiliated Hospital of Fujian Medical University, 18 Daoshan Road, Gulou District, Fuzhou, Fujian, 350001 People’s Republic of China; 3 Department of pathology, Fujian Maternity and Child Health Hospital, Affiliated Hospital of Fujian Medical University, 18 Daoshan Road, Gulou District, Fuzhou, Fujian, 350001 People’s Republic of China; 4 Department of prenatal diagnosis center, Fujian Maternity and Child Health Hospital, Affiliated Hospital of Fujian Medical University, 18 Daoshan Road, Gulou District, Fuzhou, Fujian, 350001 People’s Republic of China

**Keywords:** Congenital heart defects, Congenital heart defects

## Abstract

Early diagnosis of congenital heart disease (CHD) can improve the prognosis of neonates with CHD. We retrospectively evaluated the value of prenatal diagnosis of CHD by comparing the pregnancy outcomes. Prenatal diagnosis of CHD was established by echocardiographic evaluation of fetal heart. Amniotic fluid and/or cord blood genetic examination, pathological anatomy, casting specimen, and/or multidisciplinary-joint consultation (MDJC) were performed. A total of 1492 fetuses with CHD were diagnosed by prenatal echocardiography from 67834 pregnant women. There were 445, 236, 583, and 228 cases in groups A (simple CHD), B (simple CHD plus extra-cardiac abnormality), C (complex CHD), and D (complex CHD plus extra-cardiac abnormality), respectively. The pregnancy continuation rate in the four groups was 98.67%, 85.71%, 67.65%, and 36.84%, respectively (*P* < 0.001). The pregnancy termination rate for fetal CHD with extra-cardiac abnormalities was significantly higher than that for fetuses with only CHD (81.24% *vs*. 53.6%, *P* < 0.05). Prenatal genetic test revealed chromosomal abnormalities in 20.43% of fetuses with CHD. MDJC significantly decreased the pregnancy termination rate. In 88 cases, the original decision to terminate the pregnancy was changed after consultation and the pregnancy was continued. Of these, 87 cases culminated in live births; 65 of these children had better prognosis. Nine-segment sequential segment analysis method for prenatal fetal echocardiography was compared with the results of pathological anatomy, cast specimen, postoperative diagnosis, and postnatal ultrasound. The accuracy of prenatal ultrasound for diagnosis of fetal complex CHD and fetal simple CHD was 90.5–91.66% and 98.6%, respectively. Prenatal ultrasound is still the most effective method for fetal CHD diagnosis.

## Introduction

According to the “China Birth Defect Prevention Report (2012)”^1^, congenital heart disease (CHD) is the most common type of birth defect in China accounting for 26.7% of all birth defects in 2011. More than 130,000 new cases of CHD are reported each year in China. Globally, a total of 48.9 million cases of CHD were reported in 2015^2^. The reported incidence of CHD varies from 4 to 75 per 1,000 live births depending upon the method of diagnosis. About 0.6 to 1.9% of live born infants with CHD have moderate to severe disease^3^. CHD is the leading cause of birth defect-related deaths^4^. In 2015, an estimated 303,300 infants died around the world due to CHD^5^.

Prenatal diagnosis of CHD is an important element of antenatal care. Epidemiological studies have shown that early diagnosis of CHD and early intervention can significantly improve the prognosis of neonates with CHD^6^. Moreover, prenatal diagnosis also facilitates specific medical care immediately after delivery, which reduces the morbidity including hypoxemia, metabolic acidosis, and end-organ damage^7–11^. The purpose of this study was to comprehensively analyze the prenatal ultrasound diagnosis, genetic detection, and pathological diagnosis of fetal CHD. We assessed the pregnancy outcomes of fetal CHD and prognosis of live births; in addition, we assessed the value of multidisciplinary-joint consultation (MDJC) to provide integrated management of fetal CHD in the perinatal period.

## Methods

### Ethic statement

The personal data retrospective analysis, local pathological anatomy, and casting specimen preparation in this study were approved by Ethic Commit of Fujian Maternity and Child Health Hospital.

### CHD patients

Sixty-seven thousand eight hundred and thirty-four pregnant women who underwent fetal ultrasound examination and fetal heart screening at the mid-pregnancy stage (20–28w) between January 2012 and December 2016 at the Fujian Maternity and Child Health Hospital, were enrolled in this retrospective study. Among them, 1492 fetuses were diagnosed as CHD by echocardiography. This study was approved by the Ethics Committee of the Fujian Provincial Maternity and Children’s Hospital, and all methods were performed in accordance with the relevant guidelines and regulations. Written informed consent was obtained from the parents of the CHD fetus.

### CHD diagnosis

Fetal CHD was diagnosed according to the diagnostic criteria of the National Health Ministry’s birth defects^1^, referred to as the birth defect map, and the 10th revision of the International Statistical Classification of Diseases and Related Health Problems (ICD-10).

Simple CHD refers to heart disease with single defect, which generally does not cause hemodynamic changes. Complex CHD refers to one or more malformations of intracardiac structure and/or macrovascular structure, which cause severe hemodynamic alterations^12^.

### Classification

Based on the echocardiography examination, fetuses with CHD were classified into four groups according to the complexity of intracardiac malformation and presence of concomitant extra-cardiac abnormalities. Group A was simple CHD without any concomitant extra-cardiac abnormality; Group B was simple CHD combined with extra-cardiac abnormalities; Group C was complex CHD without any concomitant extra-cardiac abnormality; and Group D was complex CHD with concomitant extra-cardiac abnormalities. The A and B groups were defined as simple fetal CHDs, and the C and D groups were classified as complex fetal CHDs.

Based on the treatment after birth, the progeny affected by CHD were divided into surgery group and observation group.

### Fetal heart ultrasound

**Figure 1:**
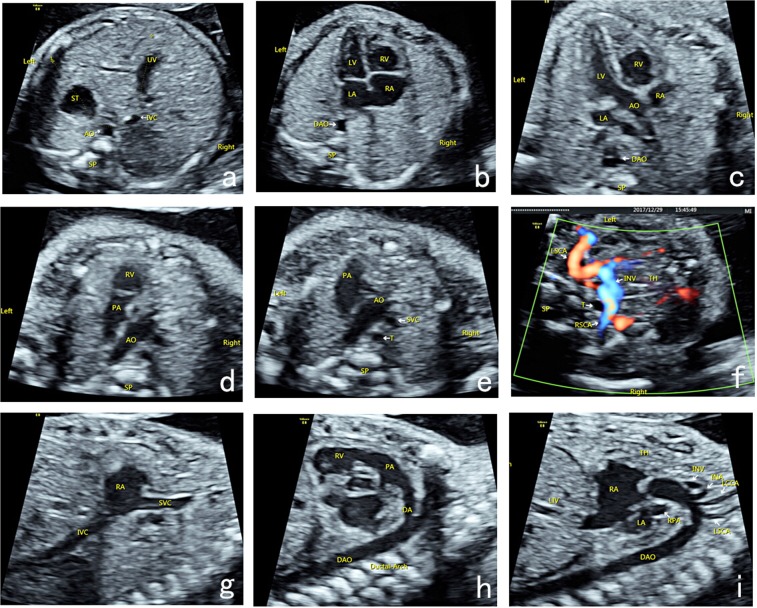
Nine-segment-sequential method for analysis of fetal heart ultrasound. Nine-segment-sequential method for analysis of fetal heart ultrasound included nine basic sections: transverse section of upper abdomen (**a**), four-chamber heart section (**b**), left ventricular outflow tract section (**c**), right ventricular outflow tract section (**d**), three-vascular-tracheal section (**e**), bilateral subclavian artery section (**f**), long axis section of superior and inferior vena cava (**g**), long axis section of ductus arteriosus (**h**), and long axis section of aortic arch (**i**). UV, umbilical vein; ST, stomach; IVC, inferior vena cava; AO, aortic; SP, spinal; DAO, descending aorta; LA, left atrium; RA, right atrium; LV, left ventricle; RV, right ventricle; PA, pulmonary artery; SVC, superior vena cava; T, trachea; TH, thymus; LSCA, left subclavian artery; RSCA, right subclavian artery; IVC, inferior vena cava; DA, ductus arteriosus; INV, innominate vein; INA, innominate artery; LCCA, left common carotid artery; LSCA, left subclavian artery.

Ultrasound examination was performed by physicians with prenatal ultrasound diagnostic qualifications using GE Voluson E8 (GE Healthcare, Lafayette, CO, USA), Philips iU22 (Philips, Copenhagen, Denmark), Siemens Acuson Sequoia 512 (SIEMENS, Munich. Germany), or S2000 (SIEMENS, Munich, Germany) Color Doppler Ultrasound with 2D/3D volume probe (frequency: 4.0–8.0 MHz). The prenatal ultrasound analysis of nine-segment-sequential method included nine basic sections (Fig. 1a–i): transverse section of upper abdomen, four-chamber heart section, left ventricular outflow tract section, right ventricular outflow tract section, three-vascular-tracheal section, bilateral subclavian artery section, long axis section of superior and inferior vena cava, long axis section of aortic arch, and long axis section of ductus arteriosus.

### Fetal CHD genetic examination

20–30 mL of amniotic fluid was collected for cell culture and karyotype analysis and prenatal single nucleotide polymorphism (SNP)-Array detection. The specimens were sent to the laboratory for inoculation, production, G-banding, and karyotype analysis using a chromosome automatic analyzer (US PE Company). The karyotype was named according to the International Cytogenetics International Naming System (ISCN). Cord blood (2.5 mL) was obtained percutaneously under ultrasound-guidance in some pregnant women for karyotype analysis and prenatal SNP-Array detection.

### Chromosomal microarray chip

Genomic DNA of the fetal amniocytes and/or the cord blood cells were extracted using the QIAamp® DNA Blood Mini Kit (Qiagen, Germany), analyzed by CytoScan 750 K gene chip (Affymetrix Genome, Santa Clara, California, USA), and evaluated using Chromosome Analysis Suite (ChAS) software (Thermo Fisher Scientific, Waltham, MA, USA).

### Fetal CHD multi-disciplinary-joint consultation (MDJC)

The MDJC team comprised of obstetricians, cardiac surgeons, geneticists, and ultrasound diagnosticians. The key tenets of the MDJC were as follows:

1. Patients in group A who showed no abnormality in genetic test were advised to continue pregnancy.
2. Patients in group B who had simple extra-cardiac malformations with no abnormality detected in genetic test were recommended to continue pregnancy.
3. Patients in group B who had complex extra-cardiac malformations or genetic syndrome tendency were recommended to continue or terminate pregnancy based on the results of genetic test.
4. Patients in group C were advised to continue or terminate pregnancy based on the evaluation of potential development trend (symptoms after birth, feasible treatment strategies, and long-term diagnosis) and genetic test.
5. Patients who had simple extra-cardiac malformations in group D were advised to continue or terminate pregnancy based on the prognostic assessment and results of genetic test.
6. Patients who had complex extra-cardiac malformations or genetic complex tendency were advised to terminate pregnancy.

### Local pathological anatomy of CHD and casting specimen

Cases wherein pregnancy was terminated due to fetal CHD underwent examination of local cardiac pathological anatomy or establishment of casting specimens. Some of the smaller cardiac specimens were observed by removing a part of the atrioventricular wall or vessel wall and opening a window to observe intracardiac morphological abnormalities. Casting specimens were established as described elsewhere^12^.

### Statistical analysis

Data analyses were performed using the SPSS statistical package version 19.0. Quantitative data are expressed as mean ± standard deviation. Qualitative data are presented as frequency (n) and percentage (%). The Chi-squared test was used to assess between-group differences, and the chi-square segmentation method was used for pairwise comparisons. The unconditional logistic regression model was used to calculate the odds ratio (OR) and 95% confidence interval (CI) of the effect of fetal CHD type and presence of extra-cardiac abnormalities on pregnancy outcomes. All *P* values were based on a two-sided test with a statistically significant level set to 0.05 (*P* < 0.05).

## Results

### Fetuses with CHD

A total of 67834 pregnant women were screened in our hospital from January 2012 to December 2016. Among these, 1492 (2.2%) fetuses were diagnosed as fetal CHD by systemic ultrasound and fetal echocardiography. The mean age of the pregnant women was 27.60 ± 3.89 years (range, 19–42); the median gestational age at the time of ultrasound examination was 24.18 ± 3.12 weeks (range, 20–28).

464 cases with fetal CHD had concomitant extra-cardiac abnormalities (464/1492, 31.1%), of which multisystem malformations (23.6%) and central nervous system malformations (22.9%) were the most common extra-cardiac abnormalities (accounting for 46.5%). The rest were edema syndrome (12.4%), skeletal dysplasia (11.2%), urogenital abnormalities (8.5%), facial abnormalities (7.8%), and digestive system abnormalities (7.3%).

### Groups of the fetuses with CHD by prenatal ultrasound

Prenatal ultrasound examination showed that 445 cases belonged to group A (including ventricular septal defect, coronary artery fistula, pulmonary artery stenosis, aortic stenosis, etc.); among these, 344 cases were followed-up, 42 pregnancies were terminated, intrauterine death occurred in two cases (all had ventricular septal defects), and two cases died in the neonatal period (ventricular septal defect and pulmonary stenosis, respectively) (Table 1). 236 cases pertained to group B (simple CHD with extra-cardiac abnormalities). In group B (222 cases were followed-up), 159 pregnancies were terminated, intrauterine death occurred in 6 cases (both ventricular septal defect), 57 culminated in live births, and three cases died during the neonatal period (two with ventricular septal defect and 1 with pulmonary stenosis) (Table 2). 583 cases were classified into group C (complicated CHD with no extra-cardiac abnormalities, including tetralogy of Fallot, double outlet of right ventricle, transposition of great arteries, hypoplastic left heart syndrome, hypoplastic right heart syndrome, heterotaxy syndrome, persistent truncus arteriosus, atrioventricular septal defect, etc.). In group C (454 cases were followed-up), 386 pregnancies were terminated, intrauterine death occurred in five cases (two cases of tetralogy of Fallot, 1 case of pulmonary atresia), 63 culminated in live birth, and 17 cases died during the neonatal period (six with coarctation of aorta and 3 with tetralogy of Fallot) (Table 3). 228 cases were classified into group D (complicated CHD with extra-cardiac abnormalities). Among these 215 cases were followed-up, 196 pregnancies were terminated, intrauterine death occurred in nine cases (four cases had atrioventricular septal defects), 10 culminated in live births, and three cases died during the neonatal period (one case each of aortic arch constriction, tetralogy of Fallot, and double outlet of right ventricle) (Table 4).

**Table 1:** Pregnancy outcomes in group A.

Type of disease	N	Valid Tracking	Termination	Intrauterine death	Delivery-survivals	Neonatal mortality
VSD	259	199	17	2	180	1
Vascular ring	125	101	5	0	96	0
PAS	47	31	17	0	14	1
coronary artery fistula	11	10	3	0	7	0
AS	3	3	0	0	3	0
Total	445	344	42	2	300	2

VSD, ventricular septal defect; PAS, pulmonary artery stenosis.

**Table 2:** Pregnancy outcomes in group B.

Type of disease	N	Valid Tracking	Termination	Intrauterine death	Delivery-survivals	Neonatal mortality
VSD	208	195	137	6	52	2
PAS	14	14	12	0	2	1
Vascular ring	12	11	9	0	2	0
AS	2	2	1	0	1	0
Total	236	222	159	6	57	3

VSD, ventricular septal defect; PAS, pulmonary artery stenosis; AS, aortic stenosis.

**Table 3:** Pregnancy outcomes in group C.

Type of disease	N	Valid Tracking	Termination	Intrauterine death	Delivery-survivals	Neonatal mortality
TOF	109	92	74	2	16	3
ASD	58	46	44	0	2	0
Heterotaxy syndrome	58	47	44	0	3	1
TGA	50	32	27	0	5	2
PA	50	42	37	1	4	1
Coarctation of Aorta	48	42	29	0	13	6
SV	34	23	23	0	0	0
DORV	33	22	20	0	2	0
HLHS	32	22	20	0	2	2
Cardiac tumor	23	17	12	0	5	0
HRHS	18	13	11	0	2	1
PTA	12	10	10	0	0	0
Ebstein anomaly	9	7	4	1	2	1
TVD	8	6	3	1	2	0
IAA	6	4	4	0	0	0
Ventricular diverticulum	5	5	3	0	2	0
PVDS	4	3	3	0	0	0
DOLV	3	2	2	0	0	0
AOPA	4	2	1	0	1	0
APW	3	3	3	0	0	0
Cardiomyopathy	3	3	2	0	1	0
Ectocardia	3	3	3	0	0	0
Ventricular aneurysm	4	3	2	0	1	0
VMD	2	2	2	0	0	0
PAS	1	1	1	0	0	0
MVDS	1	1	1	0	0	0
PVD	1	0	0	0	0	0
PVA	1	1	1	0	0	0
Total	583	454	386	5	63	17

HLHS, hypoplastic left heart syndrome; HRHS, hypoplastic right heart syndrome; PTA,persistent truncus arteriosus; TVD, tricuspid valve dysplasia; IAA, interruption of aortic arch; PVDS, pulmonary valve deficiency syndrome; AOPA, abnormal origin of pulmonary artery; APW, aortopulmonary window; VMD, valvuar mucinous degeneration; PAS, pulmonary artery sling; MVDS, mitral valve dysplasia syndrome; PVD, pulmonary venous drainage; PVA, pulmonary vein atresia.

**Table 4:** Pregnancy outcomes in group D.

Type of disease	N	Valid Tracking	Termination	Intrauterine death	Delivery-survivals	Neonatal mortality
ASD	47	43	39	4	0	0
Coarctation of Aorta	43	41	34	1	6	1
TOF	37	35	33	1	1	1
DORV	24	24	21	1	2	1
HLHS	17	17	16	1	0	0
SV	15	13	12	1	0	0
PTA	7	7	7	0	0	0
TGA	5	3	3	0	0	0
Heterotaxy syndrome	6	6	6	0	0	0
Cantrell syndrome	3	3	3	0	0	0
PA	3	3	3	0	0	0
IAA	4	4	3	0	1	0
Cardiomyopathy	2	2	2	0	0	0
Ventricular diverticulum	2	1	1	0	0	0
UAPA	3	3	3	0	0	0
Ebstein anomaly	2	2	2	0	0	0
DOLV	1	1	1	0	0	0
severe mitral stenosis	1	1	1	0	0	0
tricuspid atresia	2	2	2	0	0	0
PVD	1	1	1	0	0	0
Cardiac tumor	2	2	2	0	0	0
Coronary sinus malformation	1	1	1	0	0	0
Total	228	215	196	9	10	3

UAPA, unilateral absence of pulmonary artery.

### Genetic examination of the fetuses with CHD

Interventional prenatal genetic examination was performed for 235 (19.03%, 235/1235) cases of fetal CHD. Among these, chromosomal abnormalities were detected in 48 cases (20.43%). Of these cases, forty children had abnormal number of chromosomes, including 17 cases of 21-trisomy, 13 cases of 18-trisomy, seven cases of 13 trisomy, three cases of Turner syndrome. Other eight cases were chromosome microdeletions (three cases of 22q11.2 microdeletion syndrome, three cases of Willams-Beuren syndrome, and two cases of Cri-du-chat (cat’s cry) syndrome). The abnormal karyotype detection rate in groups B and D (39.8%) (with extra-cardiac abnormalities) was significantly higher than that in groups A and C (5.3%) (without extra-cardiac abnormalities) (Table 5). Overall, the detection rate of chromosomal abnormalities in CHD patients with extra-cardiac abnormalities was significantly higher than those without extra-cardiac abnormalities (χ^2^ = 42.375, *p* < 0.001). However, there was no significant difference in this respect between simple CHD and complex CHD (χ^2^ = 2.241, *p* = 0.134) (Table 5).

**Table 5:** The relations of chromosome abnormalities with type of CHD and extra-cardiac anomalies.

Variable	Chromosomal abnormality		*χ*^2^	*P* value
	No	Yes		
Type of CHD				
simple	112	23	2.241	0.134
complex	75	25		
Associated extra-cardiac anomalies				
No	125	7	42.375	<0.001
Yes	62	41		

### MDJC for management of fetal CHD

A total of 651 (43.6%) CHD fetuses were assessed by MDJC. Among these, 580 cases were successfully followed up. Compared with the cases that were not assessed by MDJC, the pregnancy termination rate of cases assessed by MDJC was significantly lower (Table 6), especially in groups B and C (*p* < 0.001).

**Table 6:** Differences in prognosis between fetuses with and without MDJC in each group.

Group	Consultation	N	Termination n (%)	*χ*^2^	*P* value
A	Yes	43	2 (4.65%)	2.619	0.106
	No	301	40 (13.29%)		
B	Yes	77	39 (50.65%)	25.511	<0.001
	No	145	120 (82.76%)		
C	Yes	264	207 (78.41%)	21.663	<0.001
	No	190	179 (94.21%)		
D	Yes	196	177 (90.31%)	—	0.386^a^
	No	19	19 (100%)		

Among the pregnant women who participated in MDJC, 88 pregnant women altered their original tendency (termination of pregnancy) after consultation and continued pregnancy (four cases in group A, 21 cases in group B, 45 cases in group C, and 18 cases in group D); of them, 87 cases had live births and 65 children had better prognosis (4 cases in group A, 21 cases in group B, 32 cases in group C, and 8 cases in group D).

Termination of pregnancy was recommended by MDJC in two cases affected by left ventricular dysplasia syndrome. However, the parents did not consent to termination of pregnancy and their infants died during the neonatal period. On the other hand, most pregnancies in groups C and D who continued pregnancy based on the suggestion of MDJC successfully culminated in live births in a hospital with neonatal ICU facility under the care of the MDJC.

### Survival rate of the fetuses with CHD

Four hundred and thirty fetuses with CHD were born alive; of these, 25 cases died during the neonatal period. Sixty-five patients with CHD underwent interventional or surgical treatment after birth (surgery group), while 340 patients with CHD received conservative treatment (observation group). Twenty-seven of the 65 patients in the surgery group had complex CHD. Among these, two patients were misdiagnosed by prenatal ultrasound and two cases died of pneumonia after operation. The remaining 25 cases (including two misdiagnosed cases) were followed up for periods ranging from two to five years. Four patients died during the follow-up,the survival rate of patients with complex CHD who underwent operation was 77.78%. Among the 38 patients with simple CHD who underwent surgery, three patients died of pneumonia after operation, while 35 patients survived; all of these were correctly diagnosed by prenatal ultrasound. The survival rate of patients with simple CHD who underwent operation was 92.10%. There was no significant difference in survival rate between patients with simple CHD and those with complex CHD (P = 0.101) (Table 7). Timely surgical treatment of infants with complex CHDs resulted in favorable outcomes.

**Table 7:** Comparison of postoperative survival rate of infants with complex CHD and simple CHD.

Type of CHD	N	Operative	Death	Survival rate	*χ*^2^	*P* value
Simple	357	38	3	35 (92.10%)	2.686	0.101
Complex	73	27	6	21 (77.78%)		

The observation group had 340 infants with CHD (314 simple and 26 complex CHD). Among the 314 infants with simple CHD, four of the patients had poor quality of life due to extra-cardiac abnormalities. Among the 26 patients with complicated CHD, five patients were lost to follow-up while 21 CHD fetuses were verified by final ultrasound; of these two were misdiagnosed by fetal ultrasound. These cases were not operated or were inoperable.

### Accuracy of preneonatal ultrasound for the fetuses with CHD

Based on the postoperative diagnosis of CHD in the surgery group and the final ultrasound confirmation in the observation group, the diagnostic accuracy of prenatal ultrasound for fetal simple CHD and complex CHD was 98.68% (225 of 228 from both surgery and conservative treatment groups) and 91.66% (44 of 48 from both surgery and conservative treatment groups), respectively. The diagnostic accuracy of prenatal ultrasound for simple CHD was significantly greater than that for complex CHD (P < 0.05) (Table 8). Among the misdiagnosed cases, one case was prenatally diagnosed as coarctation of aorta; however, the postpartum diagnosis showed ventricular septal defect. Another case was prenatally diagnosed as severe pulmonary stenosis; however, the suggested postoperative diagnosis was pulmonary atresia.

**Table 8:** The difference of diagnostic accuracy rate between complex CHD and simple CHD.

Type of CHD	N	ultrasound	misdiagnose	diagnostic accuracy rate	*χ*^2^	*P* value
Simple	357	228 (38 + 190)	3 (0 + 3)	225 (98.68%)	5.783	0.016
Complex	73	48 (27 + 21)	4 (2 + 2)	44 (91.67%)		

In this study population, 783 pregnancies were terminated; of these 147 cases of complex CHD were analyzed by local pathological anatomy. Twenty-one cases of complex CHD were analyzed by casting specimen. The diagnosis based on prenatal ultrasound was compared with the pathological diagnosis (as the standard) (Table 9); ultrasound diagnosis was completely consistent with the pathological diagnosis in 76.2% cases and approximately consistent with the pathological diagnosis in 14.3% cases. Thus, the diagnostic accuracy of prenatal ultrasound for complex CHD was 90.5%. In other words, the misdiagnosis rate of prenatal ultrasound was 9.5%. Analysis of the pathological anatomy indicated that the misdiagnosis of prenatal ultrasound mainly pertained to pulmonary atresia, heterotaxy syndrome, hypoplastic left heart syndrome, and valvular disease. Analysis of the cast specimens suggested that misdiagnosis of prenatal ultrasound mainly pertained to abnormal branching of aorta and abnormal venous return.

**Table 9:** Local pathological anatomy and cast specimens in complex CHD.

Type of disease	Autopsy	Casting	Exact match	Basically correct	Misdiagnosis
PS	22	2	19	3	2
Right Heterotaxy syndrome	20	2	13	6	3
Left Heterotaxy syndrome	5	1	3	2	1
TOF	26	2	23	3	2
ASD	14	2	14	2	0
Coarctation of Aorta	12	2	10	2	2
HLHS	9	2	7	1	3
HRHS	4	0	1	3	0
SV	5	1	6	0	0
PTA	3	0	1	2	0
TGA	7	2	9	0	0
DORV	5	1	5	0	1
PVA	1	0	0	0	1
Ventricular diverticulum	1	0	1	0	0
Ectocardia	3	0	3	0	0
Ebstein’s anomaly	3	0	3	0	0
APW	1	0	1	0	0
MVDS	1	0	1	0	0
VMD	2	0	1	0	1
Tricuspid atresia	1	1	2	0	0
Cardiac tumor	1	0	1	0	0
IAA	0	2	2	0	0
PVDS	1	1	2	0	0
Total	147	21	128 (76.2%)	24 (14.3%)	16 (9.5%)

### Outcomes of the fetuses with CHD

Among the 1492 cases of fetal CHD, 1235 cases (82.8%) were followed-up. Among these, pregnancy was terminated in 783 cases (63.4%) due to fetal CHD; out of these 783 cases, a pathological diagnosis was obtained in 168 cases by pathological anatomy or vascular casting. Intrauterine death occurred in 22 cases (1.8%). 430 cases (34.8%) were delivered as live births, of which 25 cases died in the neonatal period, 65 cases underwent interventional or surgical treatment, nine cases died after operation, and 340 cases were followed up.

The main causes of intrauterine death were ventricular septal defect, atrioventricular septal defect, and tetralogy of Fallot (15/22, 68.18%). The major causes of neonatal death were coarctation of aorta, tetralogy of Fallot, and ventricular septal defect (14/25, 56.00%).

The pregnancy termination rate progressively increased from group A to D (in that order) [12.21%, 71.62%, 85.02%, and 91.16%, respectively (P < 0.001)]. The survival rates of fetuses in cases of continued pregnancy were 98.67%, 85.71%, 67.65%, and 36.84%, respectively (*P* < 0.001) (Table 10). After continuity correction χ2 test treatment, the outcomes of pregnancy in the four groups were significantly different (χ2 = 697.942, *P* < 0.001) (Table 11). In addition, the Chi-square segmentation method was applied for pair wise comparisons, which indicated significant differences between groups A, B, C, and D (*P* < 0.0083 for all).

**Table 10:** Comparison of pregnancy outcomes between the four groups.

Group	Valid Tracking	Termination	Intrauterine death	Neonatal mortality	Survival	Survival rate of continued pregnancy
A	344	42 (12.21%)	2	2	298 (86.63%)	298/302 (98.67%)
B	222	159 (71.62%)	6	3	54 (24.32%)	54/63 (85.71%)
C	454	386 (85.02%)	5	17	46 (10.13%)	46/68 (67.65%)
D	215	196 (91.16%)	9	3	7 (3.26%)	7/19 (36.84%)

**Table 11:** Comparison of prognosis between the four groups.

Group	N	Termination	Intrauterine death	Survivals	Neonatal mortality	*χ*^2^	*P* value
A	344	42	2	298	2	697.942	<0.001
B	222	159	6	54	3		
C	454	386	5	46	17		
D	215	196	9	7	3		

### Risk factor analysis of the fetuses with CHD

Unconditional logistic regression analysis suggested that presence of complex CHD and extra-cardiac abnormalities were two risk factors for termination of pregnancy. Fetuses with complex CHD were at a 3.485 times higher risk of pregnancy termination compared to the fetuses with simple CHD (95% CI: 3.025–4.016). Fetuses with CHD in combination with extra-cardiac abnormalities were at a 1.935 times higher risk of pregnancy termination compared to their counterparts without extra-cardiac abnormalities (95% CI: 1.684–2.223) (Table 12). The risk of fetal death for fetuses with complex CHD was 2.925 times higher than that of fetuses with simple CHD (95% CI: 1.861–4.598). The risk of intrauterine death in case of CHD fetuses with extra-cardiac abnormalities was 3.407 times higher than that of their counterparts without extra-cardiac abnormalities (95% CI: 2.136–5.436) (Table 13). In addition, the risk of neonatal death in complex CHD was 5.154 times higher than that in simple CHD (95% CI: 3.093–8.59). Notably, extra-cardiac abnormalities did not affect neonatal death (*χ2* = 1.430, P = 0.232) (Table 14).

**Table 12:** The effect of associated extra-cardiac anomalies and type of CHD on termination of pregnancy.

Variable	Termination		*χ*^2^	*P* value	*OR* (95% *CI*)
	No	Yes			
Type of CHD					
Simple	365	201	350.219	<0.001	1.000 3.485 (3.025, 4.016)
complex	87	582			
Associated extra-cardiac anomalies					
No	370	428	92.709	<0.001	1.000 1.935 (1.684, 2.223)
Yes	82	355

**Table 13:** The effect of associated extra-cardiac anomalies and type of CHD on intrauterine death.

variate	Intrauterine death		*χ*^2^	*P* value	*OR* (95%*CI*)
	No	Yes			
Type of CHD					
simple	357	8	29.316	<0.001	1.000 2.925(1.861, 4.598)
complex	73	14			
Associated extra-cardiac anomalies					
NO	363	7	38.994	<0.001	1.000 3.407(2.136, 5.436)
YES	67	15

**Table 14:** The effect of associated extra-cardiac anomalies and type of CHD on neonatal mortality.

Variable	Neonatal mortality		*χ*^2^	*P* value	*OR* (95% *CI*)
	No	Yes			
Type of CHD					
Simple	352	5	74.800	<0.001	1.000 5.154 (3.093, 8.590)
Complex	53	20			
Associated extra-cardiac anomalies					
No	344	19	1.430	0.232	1.000 1.334 (0.827, 2.154)
Yes	61	6			

## Discussion

In this study, septal defect and conus arterial malformations were the major types of fetal CHD. The ten most common CHDs were ventricular septal defect (31.3%), tetralogy of Fallot (9.8%), vascular ring (9.2%), atrioventricular septal defect (7.0%), aortic coarctation (6.1%), heterotaxy syndrome (4.3%), pulmonary stenosis (4.1%), double outlet of right ventricle (3.8%), transposition of the great arteries (3.7%), and pulmonary atresia (3.6%), respectively. The frequency of various abnormalities is slightly different from that reported by previous studies^14,15^. In this study, we found more cases of heterotaxy syndrome, this may be attributable to the focus on comprehensive sonographic evaluation rather than the detection of a single deformity,Four structures connection in the transverse section of the upper abdomen is conducive to the rapid diagnosis of heterotaxy syndrome^13^. Increased number of the cases with vascular rings is associated with the application of bilateral subclavian artery sections^16^. Our hospital is the provincial referral centre for prenatal diagnosis and consultation. Therefore, most cases pertained to complex CHD, which may have affected the statistical distribution of the disease types.

In this study, the accuracy of prenatal ultrasound for diagnosis of fetal CHD showed slight differences from the previous reports in which the diagnostic coincidence rate was 87.89–95.35%^17–19^. Here, we performed nine-segment sequential segment analysis method of prenatal fetal echocardiography and compared with the pathological anatomy, cast specimen, postoperative diagnosis, and the postnatal ultrasound. The accuracy of prenatal ultrasound for diagnosis of fetal complex CHD and fetal simple CHD was 90.5–91.66% and 98.6%, respectively (Table 9).

Fetal CHD includes a wide variety of anomalies with varying degrees of severity, often associated with hereditary diseases and extra-cardiac malformations, or may occur as part of certain syndromes^20,21^. Although there has been considerable improvement in the accuracy of ultrasound diagnosis of CHD, it may not detect all concomitant extra-cardiac abnormalities^22^. Indeed, in our study, six CHD fetuses with simple ventricular septal defect detected by prenatal ultrasound were finally diagnosed as CHD with cleft palate after postnatal follow-up. Therefore, cases of fetal CHD should be rigorously evaluated to exclude extra-cardiac abnormalities.

The prevalence of chromosomal abnormalities in CHD patients with extra-cardiac abnormalities was significantly higher than that in CHD patients without extra-cardiac abnormalities (p ˂ 0.05), which is consistent with other reports^23,24^. Genetic testing of CHD fetus can reveal fetal genetic information, provide evidence for pregnancy decision-making, and even help guide the next pregnancy.

The complexity of CHD is significantly associated with fetal termination rates^25^. In this study, the pregnancy termination rate in cases with complex fetal CHD was as high as 87.0%. The pregnancy termination rate, fetal death rate, and neonatal mortality associated with fetal complex CHD were obviously higher than those with simple CHD (*p* < 0.05), which is consistent with a previous study^26^. The pregnancy termination rate and fetal death rate in the cases of fetal CHD with extra-cardiac abnormalities were higher than those without extra-cardiac abnormalities (P < 0.05). However, there was no significant difference in the effect of extra-cardiac abnormalities on neonatal mortality.

The pregnancy termination rates in groups A, B, C, and D showed a significant progressive increase in that order, while the pregnancy continuation rate showed a significant progressive decrease (P < 0.05). Both the pregnancy termination rate and the neonatal mortality rate in group C were higher than that in group B, which suggests that the severity of cardiac malformation has a greater impact on prognosis. This is likely attributable to the more severe disease and hemodynamic changes in cases with complicated CHD. Moreover, these cases are less amenable to surgical correction and are more likely to have impaired quality of life after surgery.

Thirty-eight patients with simple CHD who underwent cardiac surgery were followed up till date, 35 patients have shown good growth and development, while three patients died after operation, the survival rate was 92.10%. Of the 314 patients with simple CHD (A group), 310 had a good quality of life, suggesting that simple CHD is associated with a good overall prognosis. Therefore, continuation of pregnancy should be actively recommended in case of simple CHD after excluding chromosomal abnormalities and severe extra-cardiac abnormalities. Twenty-seven patients with complex CHD (group B) who accepted cardiac surgery were followed up till date, 21 patients have shown good growth and development, while six patients died after operation,the survival rate was 77.78%. In this study, good surgical outcomes were achieved in patients with tetralogy of Fallot, atrioventricular septal defect, coarctation of aorta, transposition of the great arteries, double outlet of right ventricle, pulmonary atresia, and interruption of aortic arch.

Local pathological anatomy of CHD is the most definitive method to verify the prenatal diagnosis of CHD. *In situ* observation allows for visual assessment of the cardiac morphology. Local necropsy allows for detailed assessment of the structure of heart chambers, myocardial tissue, and heart valves. Comparison of prenatal ultrasound diagnosis with results of pathological anatomy in 147 complex CHDs indicated that the misdiagnosis with ultrasound mainly pertained to pulmonary atresia, heterotaxy syndrome, hypoplastic left heart syndrome, and valvular disease; these findings are consistent with previous reports^16,24,27^.

Examination of cast specimens of 21 cases suggested that the misdiagnosis with ultrasound mainly pertained to the abnormal branching of the aorta and the abnormal reflux of the body and pulmonary veins, which is consistent with previous reports^16,28^. Local pathological anatomy can facilitate accurate post-mortem diagnosis of intracardiac malformations while cast specimens allow for assessment of the branches of the aorta and the abnormalities of body and pulmonary venous return. In addition, pathological anatomy or casting can provide real specimens for clinical and teaching purposes.

Due to the vacant positions of pediatric cardiologists in most women and children hospitals, it is difficult to accurately determine the subtype and long-term prognosis of fetal CHD, and provide authoritative treatment and prognosis counseling. Therefore, the MDJC is an effective model adopted by maternal and child specialist hospitals for perinatal management of fetal CHD. Subsequent follow-up found that there was a difference in the rate of induction of labor of the CHD patients in each group with MDJC. On comparing the pregnancy outcomes of patients with MDJC in each group, we found that the pregnancy termination rates in groups B and C were significantly lower than those without MDJC (50.65% vs. 82.76% in group B; 78.41% vs. 94.21% in group C).

In this study, the overall pregnancy termination rate due to fetuses with CHD was 64%; of them, the termination rates for simple CHD, complex CHD, and CHD with extra-cardiac abnormalities were 35.51%, 87.00%, and 81.23%, respectively. MDJC consultation and subsequent counseling of family members facilitated their understanding of the actual situation regarding the fetal CHD. Among them, 88 pregnant women and their families who participated in MDJC altered their original tendency to continue pregnancy; 65 of them achieved a better prognosis. Thus, MDJC management mode offered a distinct clinical leverage by optimizing the diagnosis and treatment of CHD.

## Conclusion

The accuracy rate of prenatal ultrasound diagnosis of complex and simple CHDs was 90.50–91.66% and 98.68%, respectively. Prenatal ultrasound nine-segment sequential analysis method is an effective method for the diagnosis of fetal CHD. Interventional prenatal genetic diagnosis to exclude chromosomal abnormalities is recommended for fetuses affected by CHD. Local pathological anatomy and establishment of cast specimens of fetal CHD are effective methods to verify the prenatal ultrasound diagnosis. MDJC significantly decreased the pregnancy termination rate. MDJC is an effective strategy for perinatal management of fetal CHD.

## Data Availability

The datasets generated during and/or analysed during the current study are available from the corresponding author on reasonable request.
